# Quality of Life and Wellbeing Parameters of Academic Dental and Nursing Personnel vs. Quality of Services

**DOI:** 10.3390/healthcare11202792

**Published:** 2023-10-21

**Authors:** Maria Antoniadou, Polyxeni Mangoulia, Pavlos Myrianthefs

**Affiliations:** 1 Department of Dentistry, School of Health Sciences, National and Kapodistrian University of Athens, Thivon 2 Street, Goudi, GR-11527 Athens, Greece; 2 Certified Systemic Analyst Professional, CSAP Executive Mastering Program in Systemic Management, University of Piraeus, GR-18534 Piraeus, Greece; 3 Department of Nursing, School of Health Sciences, Papadiamantopoulou 123 Street, Goudi, GR-11527 Athens, Greece; pmango@nurs.uoa.gr (P.M.); pmiriant@nurs.uoa.gr (P.M.)

**Keywords:** quality of life, WHOQOL questionnaire, WHOQOL-BREF version, healthcare, resilience, values, spirituality, coach, mentor, academia, academic personnel, dentistry, nursing, COVID-19 pandemic, sustainability in healthcare, healthcare sector

## Abstract

Quality of life (QOL) is based on one’s perception of one’s position in life with respect to one’s goals, expectations, standards, and concerns. It is also influenced by one’s culture and value system, workflow, and workplace situation; in turn, QOL influences the quality of service one is able to provide. In this study, we aim to report on dental and nursing academics’ QOL and wellbeing at the end of the third year of the COVID-19 pandemic. There are several studies on the impact of the COVID-19 pandemic on health professionals (nurses and dentists), but it is important to investigate their quality of life three years later; furthermore, knowledge about academic staff is very limited. The World Health Organization Quality of Life–BREF Scale (WHOQOL-BREF) tool, recording the physical, psychological, social, and environmental dimensions of QOL, was used. The WHOQOL-BREF was modified using a spiritual coaching/mentoring approach in a two-step design and validation procedure. The modified SHQOL-BREF (Spiritual Healthcare version) designed for this study was uploaded and filled in online during April–June 2023. The staff (N = 120, 75% female) of the Departments of Dentistry (44.2%) and Nursing (55.8%) of the National and Kapodistrian University of Athens participated anonymously. QOL in terms of physical health was reported at a higher level (M = 72.2 points) compared to social relationships (M = 69 points), psychological health (M = 65 points), and environment (M = 59 points) (scores reported on a 0–100 scale). Overall, QOL was rated at 66 points, while satisfaction with one’s health was at 72 points. Job satisfaction (M_1_ = 3.2) and spirituality (M_2_ = 3.0) were reported at a medium level on a five-point scale, while personal beliefs and values were reported at a high level (M_3_ = 4.0). The four areas of QOL are associated with job satisfaction, personal beliefs, and spirituality. Participant age presented a significant moderate–strong effect on physical health (F (3.97) = 2.89, *p* < 0.05, η^2^_p_ = 0.08) and on the environment (F (3.97) = 2.80, *p* < 0.05, η^2^_p_ = 0.08), and marital status had a significant effect on social relationships (F (1.97) = 9.66, *p* < 0.05, η^2^_p_ = 0.09). Married participants reported consistently higher levels of QOL compared to single participants, for all age groups. The department had a significant moderate effect on social relationships (F (1.97) = 5.10, *p* < 0.05, η^2^_p_ = 0.05), and education had a significant moderate–strong effect on psychological health (F (2.97) = 3.74, *p* < 0.05, η^2^_p_ = 0.07). PhD-level participants in both departments presented higher levels of psychological health compared to those with lower educational levels. Also, participants from the Department of Dentistry reported higher levels of social relationship QOL in all educational groups compared to the Department of Nursing. Overall, according to our findings, PhD participants generally had better psychological health. Those under 40 years of age had higher levels of physical health and environmental quality of life, while married participants and those from the Department of Dentistry had higher levels of social interactions than those from the Department of Nursing. Strategic planning on sustainability and QOL initiatives should be introduced after the COVID-19 pandemic for dental and nursing academic personnel to promote resilience and QOL scores. Enhancing the QOL of academic staff is essential for developing health promotion activities at universities and can help boost performance among staff and students.

## 1. Introduction

The WHO defines quality of life (QOL) as “an individual’s perception of their position in life in the context of the culture and value systems in which they live and in relation to their goals, expectations, standards, and concerns” [1]. As a result, QOL is a broad concept that encompasses all essential human characteristics, including both the subjective and objective assessments of the physical, mental, emotional, social, and spiritual health of an individual [2]. The individual facets of people’s perspectives on their health and wellbeing define this description. QOL has a significant impact on how a person thinks, acts, feels, and approaches challenges. It may impair one’s performance and result in job loss, which worsens one’s physical and mental health [1]. Further, effective functioning, precision, tolerance, patient care, and the satisfaction derived from work are all impacted by sleep quality, which influences the quality of life [3,4,5]. Also, there is an inverse tension in the relationship between QOL, employment, and family [6]. One’s standard of living is mostly determined by one’s financial situation and income, while health-related quality of life examines the relationship between health and quality of life, so both are concepts distinct from QOL.

As of 7 June 2023, the World Health Organization (WHO) had received reports of more than 767 million COVID-19 cases and 6.94 million fatalities since the SARS-CoV-2 virus was originally discovered in December 2019. Data from 5 June 2023, reveals that a total of more than 13.39 billion vaccine doses had been administered [7]. This pandemic’s unparalleled scope has had an impact on every element of people’s lives, encompassing their economic, emotional, and physical health. The psychological effects of the COVID-19 pandemic on healthcare professionals (HCPs) have been consistently recorded in different nations throughout the world.

Compared to the general population, patients with COVID-19 and HCPs had a higher prevalence of sleep issues [8,9]. According to HCPs, there is a significant level of perceived stigma and burnout [10], which may be a factor in the increased frequency of psychological morbidities. Additionally, it has been noted that HCPs are more likely than the general population to experience psychiatric issues [11,12]. Moderate levels of QOL (62.4%), social relationship satisfaction (42.4%), mental health (59.4%), work environment, and workplace safety perceptions (55.9%), in relation to stated quality of life, were also reported in a study by Aslanidis et al. [13], involving 170 HCPs in COVID-19 departments in Greece. According to another study in Greece, more than one-third of nurses reported having mild–severe symptoms of stress (31.4%), depression (35.4%), and anxiety (36.2%) [14]. The demanding and stressful nature of the profession can put nurses in danger and diminish their QOL. Low QOL may have an impact on the standard of care that nurses provide to their patients [15].

The continuation of COVID-19 infections following the outbreak has caused nurses to be overloaded and under a great deal of stress. These demands can be balanced by improving one’s quality of life, including one’s work [16]. While secondary traumatic stress was more prevalent in nurses with traumatic life events, compassion satisfaction was higher in those with higher scores on religion and meaning. All participants in the study by Missouridou et al. [17] viewed the change from fear to compassion satisfaction as a challenging but valuable journey. According to research, resilience may operate as a protective factor against burnout and is a strong predictor of secondary traumatic stress, burnout, and compassion satisfaction [18,19]. 

Resilience is a crucial attribute that enables healthcare professionals to overcome obstacles in the face of the COVID-19 pandemic, according to recent studies [20]. In a study of 1210 HCPs from Iran, Germany, Italy, and the Netherlands, no matter the setting, HCPs, and mainly female nurses, experienced stress and anxiety. This led to poorer sleep and decreased QOL [21]. In a cross-sectional study involving 218 HCPs, Abdelghani et al. [22] discovered that all QOL dimensions were negatively connected with health anxiety due to the COVID-19 virus. Furthermore, according to Antoniadou [23], of a study in 804 Greek dentists, men were more likely than women to experience low QOL and be dissatisfied with how their careers and personal lives were integrated during the COVID-19 pandemic. Personal resources, including close relationships with others, higher education, beliefs, and values, might act as a resilience buffer against professional challenges during times of unplanned stressful situations. Dental professionals experienced extremely high levels of physical and mental weariness, which increased by 5.5 and 8.5 times, respectively, during the pandemic [24].

Although the pandemic’s effects on health and QOL are well known [25,26], there are fewer data on how the pandemic has affected academic HCPs QOL. Overall, the healthcare academic workplace is ever more demanding, as scientific staff has clinical training, teaching, and administrative work to fulfil while technical and administrative personnel perform duties with great responsibility towards students and patients corresponding to multiple roles each day. In the third year of the COVID-19 pandemic, there is a dearth of evidence for these personnel. Thus, this study aims to investigate Greek dental and nursing academic QOL and additional wellbeing elements (working conditions, job satisfaction, personal beliefs and values, and spirituality) during the third year of the COVID-19 pandemic. The scope is to report on the relationship between sustainability in healthcare, the wellbeing of professionals, and QOL, since these terms affect productivity as well as patient care quality and safety [27,28,29,30,31]. Finally, there is a current search for resilience improvement actions based on participants’ responses that would enable them to show sustainable behaviour in future stressful events and emergency situations. Although there are studies on the impact of the COVID-19 pandemic on health professionals, there is a gap in the literature regarding academic personnel. It is also crucial to additionally comprehend how the pandemic has impacted HCPs’ QOL, the opinions of academic personnel on the “wellbeing” footprint in workflow and workspace, as well as their suggestions on the most important risk factors associated with low QOL. This research will allow the design of effective strategic resilience programs for healthcare professionals to promote their welfare and the quality of services in their line of work.

## 2. Research Methods

### 2.1. Background of the Study

**Figure 1:**
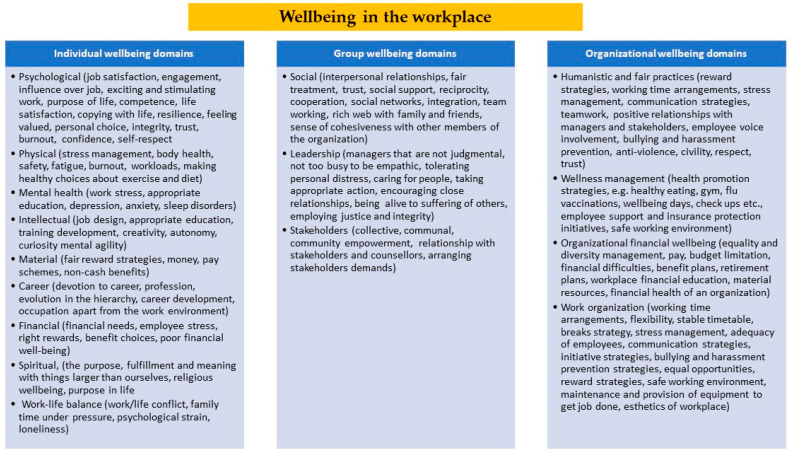
Domains of wellbeing in the workplace, according to the review analysis.

The assessment of QOL has become increasingly important in the healthcare sector, even before the COVID-19 pandemic. A related concept that has also received increasing attention lately is subjective wellbeing. Despite some commonalities, QOL and wellbeing are usually discussed in the relevant literature as separate concepts and not as synonyms. In short, QOL refers to the cognitive appraisal that a person has concerning the impact of certain factors on their daily life, while wellbeing concerns a person’s emotional response to these factors [32]. The definition and measurement of wellbeing come in many forms, have multiple dimensions, and describe certain parameters of QOL [33,34,35]. It is suggested that wellbeing at work is reflective of an overall sense of happiness and the physical and mental health of the workforce [36]. The term has three dimensions: the psychological dimension (satisfaction, attitudes, and emotions in relation to work), the physical dimension (health and safety at work), and the social dimension (interpersonal relations, teamwork, management/administrative style) [34]. Recently, a broader and more holistic explanation of wellbeing in the workplace has been developed, including sixteen individual, group, and organizational wellbeing domains [33,35,37,38,39,40,41,42] (Figure 1).

### 2.2. Identification of Factors Influencing QOL

QOL has been a key pillar in medical research for more than twenty years, supporting many clinical trials and research approaches regarding both the condition of the provider–doctor–professional in the field and the patient, and how the relationship between them can affect the QOL of both [43,44,45,46], as well as the quality of services [30]. QOL has already been explored through philosophic, psychological, and spiritual approaches on a research level through exploration of wellbeing status, as described before [47,48].

**Figure 2:**
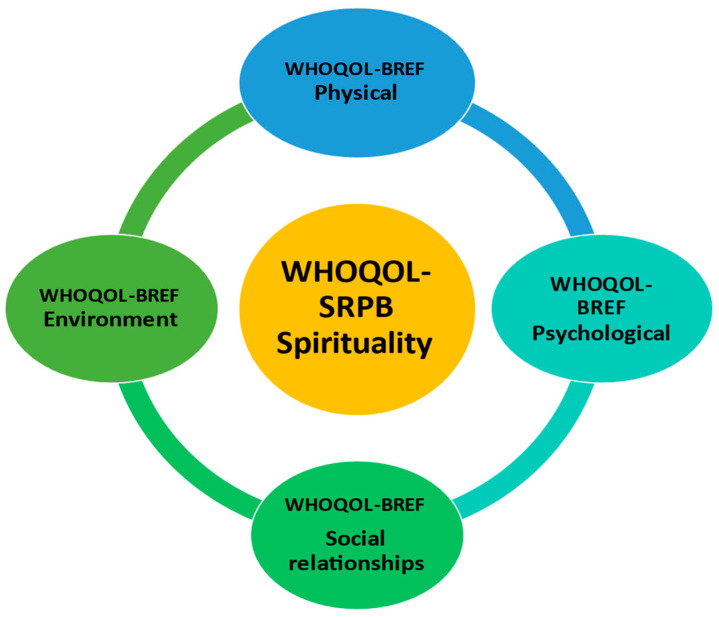
Domains of the WHOQOL-BREF (physical (pain and discomfort, energy and fatigue, sleep and rest, mobility, activities of daily life, medication, working capacity), psychological (positive feelings, thinking, memory, concentration, self-esteem, body image and appearance, negative feelings, spirituality), social relationships (personal relationships, social support, sexual activity), and environment (physical safety and security, home environment, financial resources, availability and quality of health and social care, information access, leisure, physical environment, transportation)), and the WHOQOL-SRPB (spirituality (spirituality, religion, personal beliefs)).

Of course, QOL, by definition, contains subjective elements that are not easy to capture with quantitative measurements [49]. So, there are numerous generic tools available to assess life quality. The World Health Organization (WHO) has weighted a quality-of-life assessment questionnaire (analytical and short form) in which facts and values are essentially mixed at an unsystematic level to allow some quantitative assessments of the phenomenon [50,51,52]. The WHOQOL, the tool created by the WHO, measures a variety of subjective dimensions of quality of life, estimating the complexity of the term [50,51]. The World Health Organization Quality of Life–BREF Scale (WHOQOL-BREF), a 26-item instrument, is the short, practical version of WHOQOL that records four areas of QOL (physical, psychological, social and environmental dimensions) and is officially available from the WHO in different languages [53]. The WHOQOL-BREF is one of the most well-known tools for comparing quality of life across cultures. The profile of the four WHOQOL-BREF domains is a more adequate expression of quality of life than the total score of all 26 items [51,54]. In clinical trials where brief measurements are required and in epidemiological investigations where quality of life may be one of several outcome variables, a 26-item version of the WHOQOL-BREF is appropriate. Further, we used the WHOQOL-SRPB field-test instrument (WHOQOL spirituality, religiousness, and personal beliefs (SRPB)), that is based on WHOQOL-100 questions plus 32 SRPB questions [54], addressing an additional domain of QOL, that of spirituality (Figure 2).

In this study, the English and Greek original versions of the WHOQOL-BREF and the WHOQOL-SRPB were used. This provided an opportunity to measure QOL on other conditions or with various population patterns, values, and factors. The current study had four objectives: (1) to assess whether the tool can be weighted for Greek academic healthcare professionals (dental and nursing personnel); (2) to reach an overall score for their QOL; (3) to capture and redefine factors addressed by the WHOQOL-BREF as well as to add others from the pilot study; (4) to search for the impact of these factors on the QOL of Greek healthcare academic staff; (5) to validate a modified SHQOL-BREF (Spiritual Healthcare QOL-BREF version) for healthcare professionals. 

### 2.3. Methodology of Designing the Study Questionnaire

In this study, we used a previously reliable study technique based on questionnaire data selection on QOL [52,55,56]. The technique was performed in two rounds, as has been described before [57,58]. Prior to undertaking the main data collection process, a pilot study was carried out in March 2023 on a small sample to test the preliminary questionnaire. The initial questionnaire was based on the original Greek version of the WHOQOL-BREF, in which 20 more open-ended questions were added according to the WHOQOL-SRPB and findings of previous research on the field [35,59,60,61,62,63,64] (Appendix A). The list was randomly given to ten employees from the Department of Dentistry and ten employees from the Department of Nursing of the School of Health Sciences of the National and Kapodistrian University of Athens, who volunteered to fill out the questionnaire in person within the settings of the two schools. Explanations were given when necessary. The additional questions were discussed with participants on site to determine comprehensibility, avoid biased interpretations, and promote the significance of additional factors and their impact on stakeholders in both departments. This practice is additionally suggested elsewhere [35,55].

The final questionnaire was named SHQOL-BREF (Spiritual Healthcare QOL-BREF). It investigated: (a) The demographic characteristics of the sample (Demographic Part, Q1–Q12), including gender, age, marital and educational status, type of employment, status of health (also described partly elsewhere [58]). (b) PART A (Q1–Q2): the overall QOL status and satisfaction level from health status. (c) PART B (Q3–Q9): the experiences affecting QOL. (d) PART C (Q10–Q15): the extent of importance of certain experiences in QOL. (e) PART D (Q16–Q26): the satisfaction from certain aspects of QOL. (f) PART E (Q27): the additional factors affecting wellbeing. (g) PART F (Q28–Q32): the intensity of personal beliefs and values affecting wellbeing. (h) PART G (Q33–Q38): the religious and spiritual factors affecting wellbeing. (i) PART H (Q39–Q40): the suggestions and personal opinions on ways to promote QOL and wellbeing at work and personal life. PARTS E–G (Q27–Q38) had additional questions derived from the pilot study.

The descriptive part had multiple choice answers. PARTS A–G (Q1–Q38) used a five-point Likert scale to evaluate participants attitudes and preferences for QOL and wellbeing factors used in the study. PART H (Q39–Q40) had open-ended questions where participants could fill in their proposals and express their overall view on the topic. 

The design and validity of the final questionnaire were further examined by 5 participants from each subgroup, while an independent panel of 3 experts (2 professors of the Department of Dentistry and 1 from the Department of Nursing) reviewed and revised the final version of the SHQOL-BREF questionnaire. At first, experts filled in the final version and discussed all questions with the authors in person to affirm validity. No issues regarding misconceptions of the terms or the expressions used were mentioned.

**Figure 3:**
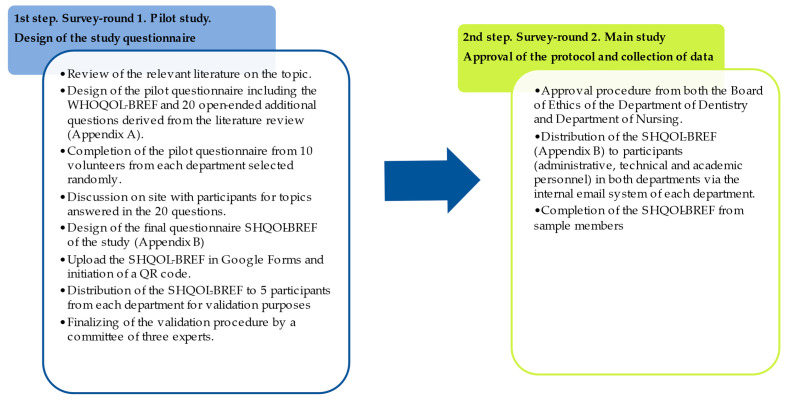
Procedures followed for the design of the study questionnaire.

The steps followed to design the SHQOL-BREF questionnaire can be seen in Figure 3.

Two categories of participants were included in the second round (main study) of the data collection process, both at the School of Health Sciences of the National and Kapodistrian University of Athens, Greece: (a) staff members of the Department of Dentistry and (b) staff members of the Department of Nursing. The sample frame was made up of the total population within the two schools, comprising 1039 employees (485 in the Department of Dentistry and 554 in the Department of Nursing), which corresponds to previous research protocols suggesting that it is possible to survey an entire population if it is of a manageable size [65]. The final e-questionnaire version of SHQOL-BREF was designed according to the previously mentioned methodology and uploaded to Google Forms. Participants could follow a specific QR code to provide direct access through smartphones. Links addressing the final questionnaire (Appendix B.2) and instructions (Appendix B.1) were then distributed to staff members through the internal email system of each department; this occurred four times in a period of one month (once per week) during April 2023. The questionnaire incorporated an introductory message describing the purpose of the study and specifically mentioning that participation was voluntary, and that confidentiality was guaranteed. Participants had the right to refuse to participate. Consent was obtained by asking participants to confirm that they agreed to complete the questionnaire by marking a “Yes, I agree to participate” box. The Board of Ethics of the Department of Dentistry (No. 547/21 November 2022) and the Department of Nursing (No. 426/9 January 2023) gave approval of the protocol. To submit the form, all questions needed to be answered. Only one submission was allowed. The questionnaires required approximately 15–18 minutes to complete. All staff members were given the same access and opportunity to complete the questionnaire. The questionnaire remained open for three months in total.

### 2.4. Statistical Analysis

The data collected from the survey were analysed using IBM SPSS v.28. Absolute and relative frequencies were calculated to summarize the demographic characteristics of the study sample. Principal Component Analysis (PCA) and Varimax rotation with Kaiser normalization were applied to group items of working conditions scaled into components. Cronbach’s alpha indices were calculated to examine the reliability of the scales and subscales of the survey. Quantitative variables were summarized with descriptive statistics (M, SD, median), and the distributions were examined in terms of normality via skewness and kurtosis [66]. Since variables were considered normally distributed, the parametric Pearson correlation coefficient was used to detect possible significance associated with QOL domains such as job satisfaction, working conditions, personal beliefs, and spirituality, whereas the combined effects of gender, age, marital status, educational level, and department on QOL domains were examined using multivariate analysis of variance (MANOVA) [67].

## 3. Results

### Sample

The sample consisted of 120 participants (75% female), working in the Departments of Nursing (55.8%) and Dentistry (44.2%). Total staff members in the department of Dentistry (485) and Nursing (554) had an overall response rate of 11.54%. Most participants (58.4%) were up to 40 years old, 45% were single, and 62.5% had no children. A proportion of 40% had a postgraduate degree, 23.3% had a PhD, 20.8% were faculty members, 56.7% were postgraduate students, and 22.5% consisted of other administrative and technical staff. Most participants reported either 1–10 years (42.7%) or 10–20 years (29.1%) of working experience. Approximately 80% of participants maintained professional activity out of the academic community, while 68.8% reported an annual family income of up to EUR 25,000. The sample was representative of all employees with respect to the demographic profile of the workforce in each department and had been described elsewhere [23,24,58]. Closed questions about sociodemographic data (e.g., gender, age, marital status, length of service, and job title) provided sufficient detail to compare the characteristics of the sample with the characteristics of the entire population of employees, as recorded by the organization’s computerized personnel system. Detailed information on demographic characteristics is presented in Table 1. 

Table 2 presents the component derived from the working conditions’ items as well as their loadings in the respective components, of the Principal Component Analysis (PCA). Three components were extracted, explaining 59.5% of the initial variability. The items “Respect from superiors”, “Respect from colleagues”, and “Possibility of temperature adjustments in the space” have been removed because they loaded on more than one component. The first component, comprising nine items, refers to working conditions and benefits, including items such as breaks for food in a suitable place, healthy meals, flexibility in working hours, babysitting, the ability to socialize with colleagues, spaces for exercise, relaxation, meditation, etc., explaining 39.6% of the variability. The second component, with 7 items, refers to work relationships, rewards, and compensation (appreciation and recognition from superiors, business relations, reward from superiors, support system from colleagues or mentors, equality in development and treatment, noise, salary), explaining 12.7% of the variability. The third component, with 4 items, refers to the workspace and the nature of work undertaken (workplace aesthetics, work creativity, feeling of giving, space comfort), explaining 7.2% of the variability. 

Descriptive statistics and reliability indicators of the study variables are presented in Table 3. QOL domain variables have been reported in 0–100 scores. Quality of life in terms of physical health was reported at a higher level (M = 72.2 points) compared to social relationships (M = 69 points), psychological health (M = 65 points), and environment (M = 59 points). Overall QOL was rated at 66 points and health satisfaction was rated at 72 points. The importance of the three working conditions’ components (working conditions and benefits, work relationships, rewards and compensation, and workspace and nature of work) presented medium–high ratings (scores between 3 and 4 on a 5-point scale). Job satisfaction was moderate (M = 3.2 points in a 5-point scale), and spirituality was reported at a medium level (M = 3.0 points in a 5-point scale). Personal beliefs and values were reported at a high level (M = 4.0 points in a 5-point scale). 

The Pearson correlation coefficients between the study variables are presented in Table 4. QOL in terms of physical health was positively correlated with job satisfaction (r = 0.377, *p* < 0.01) and personal beliefs (r = 0.332, *p* < 0.01). Moreover, psychological health was associated with higher levels of personal beliefs (r = 0.595, *p* < 0.01) and spirituality (r = 0.262, *p* < 0.01). Social relationships were positively related to workspace and nature of work (r = 0.293, *p* < 0.01), job satisfaction (r = 0.319, *p* < 0.01), and personal beliefs (r = 0.304, *p* < 0.01). Environmental QOL was related to higher levels of job satisfaction (r = 0.504, *p* < 0.01), personal beliefs (r = 0.282, *p* < 0.01), and spirituality (r = 0.223, *p* < 0.01). Overall, our findings show that the four areas of QOL are associated with job satisfaction, personal beliefs, and spirituality; so, the higher these are, the better the quality of life in those four domains. Also, the dimension of the workspace and the nature of the work were found to be positively associated with the quality of social relations, i.e., in a creative environment where people feel that they offer themselves and are accepted, there is better quality of social relationships.

The MAVONA results of the tests of the between-subject effects of gender, age, marital status, department, and education level on the four QOL domains are presented in Table 5. MANOVA was chosen to account for the multivariate effects of demographic and job characteristics on QOL domains. Significant effects were detected for age, marital status, department, and education. Participants’ age presented a significant moderate–strong effect on physical health [F (3.97) = 2.89, *p* < 0.05, η^2^_p_ = 0.08] and environmental QOL [F (3.97) = 2.80, *p* < 0.05, η^2^_p_ = 0.08]. Marital status presented a significant moderate–strong effect on social relationships [F (1.97) = 9.66, *p* < 0.05, η^2^_p_ = 0.09].

**Figure 4:**
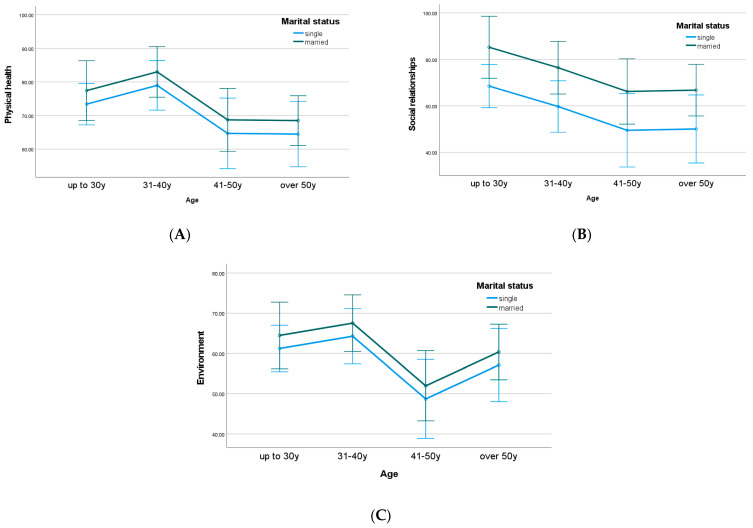

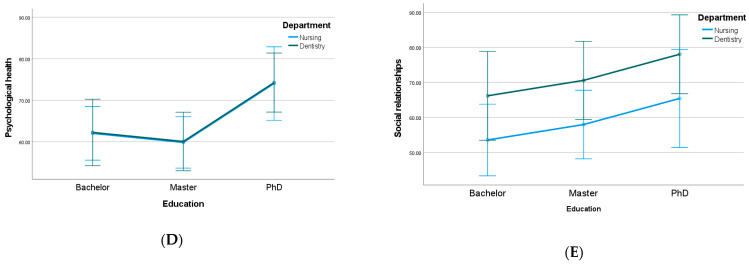
Estimated marginal means of the MANOVA model for the domains of QOL: (**A**) physical health against age groups and marital status; (**B**) social relationships against age groups and marital status; (**C**) environment against age groups and marital status; (**D**) physical health against department and educational level; (**E**) social relationships against department and educational level.

Specifically, as presented in Figure 4A, both married and single participants under 40 years of age reported higher levels of physical health compared to older participants. In terms of social relationships (Figure 4B), married participants reported consistently higher levels of QOL compared to single participants for all age groups. Despite the non-significant differences between marital status groups, participants under 40 years old reported higher levels of environmental QOL compared to older participants (Figure 4C). 

In addition, the department also had a significant moderate effect on social relationships [F (1,97) = 5.10, *p* < 0.05, η^2^_p_ = 0.05] and education had a significant moderate–strong effect on psychological health [F (2,97) = 3.74, *p* < 0.05, η^2^_p_ = 0.07]. As presented in Figure 4D, PhD level participants in both departments presented higher levels of psychological health compared to lower educational levels. Also, Figure 4E shows that participants from the Department of Dentistry reported higher levels of social relationships and QOL in all educational groups compared to the Department of Nursing.

Almost half of the participants, a percentage of 57.5%, gave a positive answer on Q28: “To what extent do you think a coach/mentor/spiritual guide would help you in your self-awareness and development?” (27 people answered, “A moderate amount”, 27 “Very much”, and 15 “an extreme amount”). Data from the open-ended question Q39 “What would you like to change in your workplace to be more satisfied?” revealed that 29.2% of the participants reported changes in administrative issues (stable timetable, breaks, organization, change position, respect the rights of employees, evolution in the hierarchy), 25% of them would expect better salary, 24.2% would rather experience better behaviour from superiors (respect, recognition, reward, equality, meritocracy, justice and common values), 17.5% would prefer better staff relations (team working, responsibility, respect), 14.2% would appreciate an increase in staff employment, and 13.1% discuss improvements in aesthetics and infrastructure (improvement of space, equipment, privacy, noise hygiene, software). Only 2.5% discussed education on site and 1.6% mentioned other practical issues such as distance from home and transportation issues.

From question Q40 “What would you like to change in your life to be happier?”, most of the participants (48.3%) would prefer sufficient personal time to relax, practice gymnastics, travel, spend time with friends and family, take up hobbies, or pursue further education. Only 10.8% of them would rather change personality issues (be more positive, open, and resilient, be less of a thinker, reduce stress, change worldview). Further, 10% of them would prefer to resolve economic issues to be happier and 7.5% would like to improve human relations (intimate and friendly). Additionally, 6.7% of the participants would like to solve home issues (residence, house, distances), 5.8% of them would rather improve health issues to be happier (health, body image, healthier food, access to health modalities), and 5.8% would prefer to improve work conditions (stable timetable, relevant position to education, no work except of working hours (e.g., on Saturdays and Sundays, etc.). Finally, 3.3% suggest improvements in security issues and 3.3% discuss societal issues (environment, state status, social profile). 

We could mention further some written approaches of the participants in the pilot and main study that give their overview of the topic. “There is a need to improve working conditions at all levels: Quality, safety, ensuring necessary resources for adequate operation, personnel, hygiene. Retraining, ensuring sufficient staff for the services, improving bureaucracy, effective management systems of an organization by ensuring people related to the subjects they are called to manage, rationalization of division of labor, opportunities for the promotion of qualifications, benefits to employees, etc.” and “well-being for me is to be happy most of the day… and in order to be happy, I need time for myself, qood people around me to work or chat, respect and reward at work, a family dinner without stress, time with friends and money to spend in order to be educated, travel, enjoy nature and silence!…”

## 4. Discussion

In this study, data on the QOL and wellbeing of a sample of Greek dental and nursing academic personnel, three years after COVID-19 was declared a pandemic, are presented. To our best knowledge, this is the first study to evaluate QOL among academic personnel. In Greece, quality of life and presence of negative emotions among frontline healthcare workers have been assessed [13,23]. The overall QOL was rated in accordance with 66 points in this study. The importance of the three components of work conditions presented medium–high ratings. The level of physical health was reported to be higher (M = 72.2) compared to social relationships (M = 69), psychological health (M = 65), and environment (M = 59). Similarly, in the study conducted by Ghazy et al. [68], investigating healthcare professionals’ QOL two years after the COVID-19 outbreak in Arab nations, the highest mean score was in the physical domain (M = 68), indicating adequate energy, the capacity to deal with fatigue, pain, and discomfort, as well as adequate sleep and rest. Previous studies have also shown the association between pre-existing medical issues (negative self-perceived health status or being diagnosed with systemic illnesses) and poor physical health QOL [25,26,69]. Chronic diseases may significantly affect one’s ability to participate in the workforce owing to physical, emotional, or social problems [70], and chronic pain or illnesses are associated with reduced compassion, satisfaction, absenteeism, and presenteeism [71]. In our study, most participants (58.4%) were below 40 years old, and both married and single participants under 40 years of age reported higher levels of physical health compared to older participants, thus explaining the overall QOL of this study.

### 4.1. Physical Health Dimension and QOL

Physical health QOL was rated at 72 points and positively correlated with job satisfaction and personal beliefs, meaning that professionals that are satisfied with work and have a stable value system feel physically healthy. Quality of life in terms of physical health was reported at a higher level than all other QOL dimensions (social relationships, psychological health, and environment), suggesting that physical health is a clear frontrunner in importance for QOL among the sample of this study. Only 5.8% of our participants would like to make changes to their health issues to be happier. In the study conducted by Kua et al. [72], where healthcare workers in the second year of the pandemic experienced changes in their working hours and the majority had reduced their physical activity frequency (42.5%) and duration (42.8%), 29.4% reported that their physical wellbeing had been negatively affected. This fact, though was observed during the first year of the pandemic, when most of the participants were reporting health issues [73]. Of course, except for the pandemic, there are other chronic health problems not directly attributed to the pandemic; these mainly comprise ergonomic working conditions that can, in different ways, affect the general health of healthcare professionals [74]. These differences during the pandemic could be attributed to our sample, to the gradual return to normality during the period of our study, and to the unevenness of our sample, consisting not only of clinicians but also of administrative personnel. Our data showed that age also affects physical health QOL; this is in agreement with findings that suggest that ageing nursing personnel face more health issues compared to physicians due to labour-heavy physical tasks [75]. In contrast, age was not found to be a significant factor in the Ghazy et al. [68] study which sought to determine a physical activity score. Meanwhile, in the study conducted by Hawlader et al. [76], people over 45 years of age have a 52% lower likelihood of being in good physical condition; this was the case in our study too. 

### 4.2. Psychological Health Dimension and QOL

Our data reveal that psychological health was associated with higher levels of importance of personal beliefs and spirituality, while education had a significant moderate–strong effect on psychological health. Education is increasingly being recognized as a crucial component in each nation’s economic engine, in terms of both training and development. Human capital theory, a well-known economic rationalist approach, emphasizes the benefits of investing in education: education and training (human capital) raise worker productivity, which raises the value of educated people [77]. People who spend time, effort, and money to their education do so with the hope of building a better career and increasing their lifetime earnings. The assumption that educational indicators, such as enrolment rates and average scores on standardized achievement tests, are also social indicators or markers of the distribution of living conditions within a society comes from the idea that education is essential to one’s quality of life. 

Furthermore, spirituality can be defined as follows: “a dynamic and intrinsic aspect of humanity through which individuals experience relationship to themselves, family, others, community, society, nature, and the significant or sacred and seek ultimate meaning, purpose, and transcendence” [78]. Beliefs, values, customs, and practices are ways in which spirituality is expressed. For many people, spirituality may offer a way to discover (or rediscover) meaning in the face of stressful life events and is important for healthcare professionals as well as patients since it may have an impact on their psychological health and the way they live and practice. In our study, the psychological health was associated with higher levels of personal beliefs and spirituality not discussed elsewhere.

Marital status can also enhance psychological health. Our findings correspond to findings from the study of Suryavanshi et al. [79]; the study demonstrated a high prevalence of symptoms of depression and anxiety and low QOL among Indian HCPs during the COVID-19 pandemic. Also, in that study, combined depression and anxiety were found to be 2.37 times higher among single HCPs compared to married HCPs. Overall, our findings correspond to findings of other studies too, demonstrating a high prevalence of symptoms of depression and anxiety and low QOL among healthcare professionals working with COVID-19 patients; here, gender, marital status, and age were found to significantly influence depressive symptoms, posttraumatic stress symptoms, and psychological distress [24,80].

Further, it has been mentioned that, during the pandemic, factors associated with psychological distress among dentists were lower income, burnout, high job stress, career-choice regret, and lack of sufficient personal time. For dental nurses, these factors were found to be age, lower income, longer working hours per week, burnout, high job stress, low job satisfaction, lack of sufficient personal time, and a poor medical environment [80]. In our study, though, lack of sufficient personal time was reported by 48.3% of our participants as a basic factor in low psychological health. Further, being female and not in a relationship were found elsewhere to be associated with higher levels of depressive symptoms, whereas being female and older was found to be related to higher levels of posttraumatic syndrome [81], as was the case in our study too. Also, in the dental population, male professionals were overwhelmed with psychological problems more than their female colleagues during the pandemic due to the reversing of roles in their routines [24]. In this study, gender is not directly correlated with QOL, possibly due to the different job distributions among academic personnel, the distance working during the pandemic, and the overload of academic work for all participants. 

### 4.3. Environmental Dimension and QOL

The three working conditions’ components revealed by our analysis (working conditions and benefits, work relationships, rewards, and compensation, and workspace and nature of work) are of medium–high importance for environmental QOL in our sample. Environmental QOL consists of a variety of different relationships and working conditions in the workplace as suggested also elsewhere. In the study by Omidi et al. [82], nurses who worked more than 36–40 h a week had a poorer score in the environment category. The environmental domain findings covered topics including physical security, leisure time activities, and transit options. In the Ghazy et al. study [68], low mean scores were similarly found for the environmental and psychological domains, indicating poor home environment satisfaction, poor participation in leisure activities, comprised activities of daily living, general law and order situations, reduced mobility and greater discomfort, fatigue, and reduced work capacity. In our study, though, environmental QOL was related to higher levels of job satisfaction, personal beliefs, and spirituality, meaning that a strong value and existential base could go hand in hand with environmental QOL. Although it is not specified whether these bases initiate a higher environmental QOL or whether it is a good environmental QOL that boost values and spirituality, the correlation among these parameters is not to be denied. Other researchers agree that feeling comfortable at work is associated with a positive perception of the supportiveness of the organizational climate, since insecurity is a key dimension of negative wellbeing and low quality of services [83]. If the work environment is not exciting, fulfilling, rewarding, stimulating, or enjoyable [38], there is not a compassionate culture that demonstrates support, care, and empathy. Further, where there is no promotion of employees’ wellbeing, people become disappointed and even angry. Their performances reduce in quality, and the quality of the services are thrown into question [35]. On the other hand, people who feel well and valued perform better than those who feel rejected [84]. We should further mention that when people feel that they have been unfairly treated, they experience a series of injustices. They are frustrated because feelings of inferiority preoccupy the mind and dominate one’s thoughts [85]. Perceived unfairness is a significant demotivator [86]. Optimistically, however, only 24.2% of our sample reported that they would rather experience better behaviour from superiors (respect, recognition, reward, equality, meritocracy, justice, and common values), and only 17.5% reported that they would prefer better staff relations (teamwork, responsibility, respect); these results possibly show that such issues are not especially prevalent in either of the departments. So, high-quality relationships between employees and counsellors, mentors/coaches, and customers are important for employees’ wellbeing at work [33]. Employees report that they would want to be informed about ethical practices and corporate social responsibility, which includes fairness, compassionate leadership, and fair work practices [35], to perform better in their positions. In our study, only three employees reported feeling that their role was irrelevant in any way, or the need to change position for such reasons. Of course, we must consider that this study has taken place in a public university where people are being paid a fixed amount by the state; most of them have a permanent position in the organization, which at least should dispel any doubts around job security. 

Working conditions concerning hours of work, timetable, and breaks seem to also be significant factors influencing QOL in our sample. This means that not having adequate staff, and thus not having time for breaks and being overloaded with tasks, has a negative impact on care, teaching, and administrative or technical support quality and services, as has been discussed by other researchers [86]. It has been reported that low nurse staffing levels in local care settings are associated with poorer user experiences [87], hospital readmissions [88], and reduced continuity of care [89,90]. On one hand, we must consider the number of staff and the distribution of staff groups (staff ratio); on the other hand, we must consider the combinations of skills necessary to provide the care that is needed in these specific settings [91,92]. In our study, 14.2% of the participants commented on the staff ratio adequacy, and 5.8% of them discussed the right position according to education and skills. As suggested elsewhere, not being in the right position, being overwhelmed with tasks and working overtime due to inadequate staffing results in high levels of sickness, absence, and part-time work or retirement among dentists [23,24]. Long hours of working were reported to comprise an important factor in our study too; as has been reported previously, such conditions led to a drastic increase in burnout among dentists and nurses before and during the pandemic [23,24,93,94,95,96]. These conditions were found to have a negative impact on employees’ health fatigue and relationships, resulting in absences, more accidents and errors, reduced satisfaction, low psychological wellbeing, and doubtful job performance [97,98,99,100]. 

Finally, work–life conflict is mentioned as a negative factor for QOL and wellbeing. In the relevant literature, it is not unusual to encounter reporting of significant need for personal time among employees in the healthcare sector [68,84]. The fact that most individuals are continuously striving to balance their work and personal lives is a big challenge at present [101]. In the study conducted by Shivakumar and Pujar [97], approximately 40% of the staff expressed that they were unable to spend enough time with family, while most staff agreed that there should be flexible working hours and compensatory holidays to assist them in maintaining a good work–life balance. In our study, 48.3% of our participants reported a need for more personal time and free time to spend with family and friends; participants additionally reported a desire for flexibility in the timetable, and an avoidance of working overtime on Saturdays and Sundays. This situation can lead to reduced commitment, discipline, and performance, as has been discussed [95]. Also, in our study, 5.8% of the employees reported a desire for changes in administrative proceedings concerning working hours. Employees working overtime experience negative work–life balance, health issues, and family disturbances that ultimately lead to demotivation and increased staff turnover. Moore [102] has indicated that those employers who can provide supportive policies to facilitate good long-term work–life balance and create a culture where employees are positive, productive, and loyal will be regarded as “good cases” for workplaces to refer to.

Additionally, participants under 40 years of age reported higher levels of environmental QOL compared to older participants, signalling that millennials and Gen Z populations give more importance to natural environments and aesthetics in the workplace, as has been discussed [103]. In the study conducted by Schell et al. [103], “a high rank” was found among reported desire for aesthetic improvement; these were associated with psychologically demanding work, negative work stress, sleep disturbances, problems at work, musculoskeletal pain, and lower age. We did not find such a correlation, with only 13.1% of our participants worrying about aesthetics, infrastructure, equipment, and maintenance. Of course, environmental QOL comprises more factors than just aesthetics. It also discusses the matter of privacy in the workplace, which is another important issue associated with the concept of confidentiality [104]. Privacy refers to the right to be left alone, free from intrusion, having an independent workspace, including the right to make independent decisions based on personal beliefs, feelings, or attitudes; the right to control body integrity; and the right to decide when and how sensitive information is shared [104,105,106]. Only two participants in our sample reported a desire for improved privacy in the workplace, possibly because privacy is well-controlled in both departments. Noise and hygiene issues were mentioned by only four people in our study as negative factors affecting QOL and wellbeing, as has been discussed elsewhere [107]. 

Environmental QOL is further related to the financial issues of both individual employees and the organization itself. It is mentioned that the environment in which individuals live and work has a major and significant influence on how individuals respond to their own feelings of wellbeing [84]. So, an emotionally healthy workplace is financially sound, has improved potential for employees to flourish, offers better services, enhances motivation and effort, and can afford to be optimistic about its future [108]. This has a serious effect on employees, as in our case, with more than 50% of our participants discussing a need for certain organizational changes that can affect the economic status of employees in both departments (better salaries, adequate staff). If the financial wellbeing of an organization is not healthy and administration and managers need to function with limited budgets with a mindset of reducing expenditures, then there are implications: job insecurity, increased workloads, job-related stress and strain [35]. Then, the success of an organization is judged based on its financial performance and its ability to provide high-quality goods and services over time [30,109]. A joy-filled workplace is said to improve financial performance and ensure positivity and meaning in the work completed [110], thus improving QOL and quality of services. This should be an administrative strategic plan for both departments included in our study.

The concept of work–life integration, or the productive mixing of ones’ personal and professional duties, is gaining popularity [111]. According to this viewpoint, employment is but one part of our lives that must be considered alongside other crucial issues like our relationships with our homes and families, our communities, and our own personal wellbeing. Work–life integration resembles a Venn diagram of overlapping interests more than a scale with two opposing sides. Consequently, to achieve a good work–life balance, academic employees must carefully consider their priorities and ambitions, as well as their goals by evaluating the importance of tasks, managing time and procrastination, setting boundaries, and refining the process periodically.

### 4.4. Social Relationships Dimension and QOL

A significant relationship between marital status and QOL was reported in the study conducted by Han et al. [112], with this relationship to be affected by gender and age. Further, in the study conducted by Puciato et al. [113], there was a dependence between marital status, perceived health condition, and QOL. Mmale sex, higher education, being an entrepreneur, college student, or white-collar worker, and good financial status were associated with the highest assessments of QOL and perceived health conditions [113]. In our study, although married participants reported consistently higher levels of QOL compared to single participants, there were no differences among age groups or sex. In contrast, in a sample of Greek dentists during the pandemic, QOL was significantly lower for men than women [23]. Further, in our study, dental academic personnel reported a better QOL in social relationships than the nursing personnel. 

The “Harvard Study of Adult Development” that started in Boston in 1938 and followed 2000 volunteers for 85 years, covering three generations (grandparents, parents, and children), revealed that the happiest people undertook two main occupations during those 85 years: taking care of their health and cultivating meaningful relationships with others [112]. This takeaway has been suggested by others [111,112,113,114,115,116,117] and is supported by our findings. 

### 4.5. Proposals on Academic QOL

Initiatives to enhance QOL and wellbeing should be embraced by organizations as a priority; otherwise, they could be left behind in the future. Healthcare facilities that foster wellbeing are perceived as employers who conduct “best practices” and are recognized by current and prospective employees as offering a desirable place to work [30,34]. There is a universal agreement that the healthcare industry is one of the most hazardous environments to work in. Employees in this industry are constantly exposed to a complex variety of health and safety hazards, (e.g. noise), constant human contact, ergonomic issues, and standing for long periods of time. Long working hours, work overload, and shift work add to these stressors [118]. Our findings agree with the description of this hard-working environment [17,119] and changes should be incorporated into future administrative plans. According to participants’ suggestions, the timetable should be reorganized to fulfil a stable but flexible timetable of morning shifts (8.00–16.00 h), lunch breaks, avoiding overtime and weekend work, and having access to healthy food. Spaces for relaxation, gyms, and meditation should be designed in the facilities of both departments. Communication and collaboration should be enhanced to cultivate trust. International collaborations could further reinforce the openness of the departments and bring a sense of creativity, excitement around learning, and meaningful working mindset to employees. Finally, periodical educational seminars on resilience themes should be incorporated for all personnel. The role of the employee’s coach or mentor within both departments should be enhanced, as more than 55% of staff members would like a coach’s assistance to further help them in their self-awareness effort and self-development. This has been suggested before [120]. Overall, it seems from our findings that professional success alone does not guarantee happiness, although it can be enjoyable. The study revealed that happier people were not isolated. In fact, the happiest people valued and nurtured human relationships. Education and cultural awareness levels, which tended to be higher among those with higher salaries, have been shown to be important factors in adopting healthy habits (promoted more frequently since the 1960s) and in ensuring better access to healthcare. Loneliness, which is becoming increasingly frequent, causes anxiety [79,121,122,123]. Cultivating, strengthening, and expanding human relationships—in fact, maintaining social ties, which, just like fitness, also requires constant practice—is also proposed. Friendships and relationships in academia require regular commitment: even a simple phone call can prevent them from slackening. Engaging in activities that bring joy and encourage companionship, such as sports, hobbies, and volunteer work, can broaden one’s network of relationships, especially among academic personnel who do not have the time to expand their relationships outside of academia. The truth is that no one’s life is free from difficulties and challenges; however, social skills can contribute to resilience. The administrators of the two departments, especially the Department of Nursing, should encourage social events where all personnel can access and enjoy shared leisure time.

### 4.6. Limitations and Benefits of the Study

This study has some strengths and some limitations. First, the data were directed at specific healthcare sectors of a public university, making it less possible to understand the QOL and health of individual professionals or establish health policies based on generalized evidence. Above all, these data reflect the experiences of academic personnel in only one institution, which is in a high-ranking position (NKUA is ranked 252nd worldwide among 12,000 universities, 55th among the 3465 universities of the European Union, and 1st in Greece among the Greek universities included in the ranking) [124]. The QOL among staff members does not reach a high level (less than 80 points). Further estimation of values, culture, and regions or work positions could bring about improvements in QOL, as has been suggested elsewhere [122]; data can provide better insights into the correlations between academic effort and willingness to promote QOL with physical, psychological, and mental health environments and working/administrative issues. Additionally, the study sample should be augmented in future studies to allow for a wider simulation of the respective population. The unwillingness to participate in this study is relevant to behaviour reported elsewhere [58] and could be addressed through personal interviews in the future. 

This study was cross-sectional in nature; hence, there are limitations to interpreting the causal relationships between certain demographic characteristics and QOL or specifying the physical health disorders that are involved in the estimation of health QOL; this is due to the generic nature of the information collected, as is the case elsewhere [75,125]. The questions were asked at the current time, and the question about QOL would inevitably be reflective of the day on which the participant filled out the questionnaire. Therefore, we believe that we are working with data of a one-way relationship. Thus, it is unlikely that the participants’ previous QOL resulted in the current estimation of factors addressed here. Further, to measure the relationship between QOL, work wellbeing, demographic characteristics of healthcare academic personnel, and quality of services more accurately, other issues must be considered. For instance, more studies should be conductedabout the following: the positive impact of marital satisfaction and educational and economic status on the quality of life of academic personnel; the quality of human relationships at work in accordance with gender or age group; the increase in wellbeing due to ethical, religious, and spiritual values and practices such as regular breaks, gym exercise, or meditation on the work site. Privacy and its various dimensions in the occupational health context should also be examined in connection with QOL, as suggested elsewhere [104,106]. 

Despite these limitations, and to the best of our knowledge, this is the first study on the relationship between QOL, demographic characteristics, wellbeing factors, and healthcare personnel in a public academic environment—specifically, between two different healthcare departments. Further estimation of QOL in other departments could give more information to design resilience strategies for future emergency situations. Finally, the SHQOL-BREF, although validated in our study, could be further tested in other public or private healthcare departments. 

To gain a deeper understanding of the relationship between QOL and wellbeing elements across time, it would be ideal to adopt a longitudinal approach in future studies. Two academic departments from one Greek institution were the subject of our sample. Therefore, researchers must consider various professional categories and nations in future research. Moreover, future research should explore the role of resilience in QOL and job satisfaction, and more attention should be paid to the role of spirituality.

Our study has theoretical and applied implications. From a theoretical perspective, this study offers important insights into the wellbeing and quality of life of academic health professionals in Greece. Learning more about the connections between QOL, job satisfaction, and spirituality among academic staff in higher education can help us better understand how working conditions affect employees and guide the development of new strategies that will enhance both academic staff wellbeing and educational quality.

This study clarified the benefits of supporting the elements that are significantly and positively associated with QOL from a tactical standpoint. Governments and university boards can take action to improve aspects of QOL and wellbeing and prevent burnout and, as a result, reduce intention to quit, absenteeism, presenteeism, and occupational health conditions by understanding how a phenomenon may operate in a particular profession. Policies that support the career development of academic personnel and effective strategic resilience programs for all healthcare professionals are needed and should be designed and implemented accordingly.

## 5. Conclusions

In this study, a modified SHQOL-BREF (Spiritual Healthcare version) was administered to personnel of the departments of Dentistry and Nursing of the National and Kapodistrian University of Athens three years after the onset of the COVID-19 pandemic. Overall, QOL was found to be rated at a medium–high level. Physical health was found to be rated as having the highest QOL of the four domains, while environmental factors had the lowest. Higher levels of QOL were correlated with marital status, a PhD education, and young age (under 40). Social interactions were also somewhat influenced by the respective departments. It is indicated that the four dimensions of QOL are related to three factors: job satisfaction, personal beliefs, and spirituality. The better these three factors are, the higher the quality of life is in those four categories. Additionally, the dimension of the workplace and the nature of the work are positively correlated with the calibre of social connections, i.e., social relationships are of higher calibre in a creative environment where people feel as though they can contribute and are accepted.

Understanding the role that sociodemographic, individual, interpersonal, and workplace traits play in staff members’ quality of life, overall wellbeing, and academic performance is crucial. The QOL delivers creative workflows that impact both the performance of the person and the entire organization. In addition to being crucial for creating health promotion initiatives at universities, enhancing the QOL of academic staff can also improve staff and students’ performance. After the COVID-19 pandemic, dental and nursing academic staff should begin strategic planning on human sustainability and QOL activities to foster resilience and QOL scores.

## Figures and Tables

**Table 1:** Demographic characteristics of the study sample (N = 120).

		Ν	%
Gender	Male	30	25.0%
	Female	90	75.0%
Age	Up to 30 years old	41	34.2%
	31–40 years	29	24.2%
	41–50 years	18	15.0%
	51–60 years	24	20.0%
	61 years and above	8	6.7%
Marital status	Single	54	45.0%
	Living like married	11	9.2%
	Married	49	40.8%
	Widowed	1	0.8%
	Divorced	5	4.2%
Number of children	No children	75	62.5%
	1–2 children	43	35.8%
	3–4 children	2	1.7%
Education	Secondary (high school, technical school)	9	7.5%
	Tertiary (Bachelor)	35	29.2%
	Postgraduate (Master)	48	40.0%
	Ph.D.	28	23.3%
Job position	IDAX administrative staff (administrative)	8	6.7%
	Administrative staff IDOH (administrative)	7	5.8%
	Research Associates (Unpaid) (scientific)	6	5.0%
	Research Associates (paid) (scientific)	2	1.7%
	Faculty members (scientific)	25	20.8%
	Postgraduate students (scientific)	68	56.7%
	EIB staff (technical)	1	0.8%
	Technical staff IDOH (technical)	3	2.5%
Work experience	1–10 years	50	42.7%
	10–20 years	34	29.1%
	21–30 years	20	17.1%
	Over 31 years	13	11.1%
Department	Nursing	67	55.8%
	Dentistry	53	44.2%
Non-academic professional activity	Sole proprietorship with more than 2 employees	6	5.0%
	Sole proprietorship	5	4.2%
	Sole proprietorship with 1 employee	11	9.2%
	I do not know/I do not answer	17	14.2%
	I do not maintain a professional activity outside of academic hours	26	21.7%
	Company (member or director)	9	7.5%
	Hospital employment	40	33.3%
	Provision of services without an individual seat	6	5.0%
Annual family income	Under EUR 15,000	47	42.0%
	EUR 15,001–25,000	30	26.8%
	EUR 25,001–50,000	25	22.3%
	EUR 50,001–100,000	9	8.0%
	EUR 100,001 and above	1	0.9%

**Table 2:** Working conditions item loadings in the respective component of working conditions.

	Component		
	Working Conditions and Benefits	Work Relationships, Rewards, and Compensation	Workspace and Nature of Work
Possibility of a half hour break for food	0.867		
Possibility of a break in a suitable place	0.845		
Possibility of receiving healthy meals	0.744		
Flexibility of working hours	0.727		
Possibility of babysitting	0.668		
Working hours compliance	0.616		
Possibility of continuing education in the same area	0.607		
Ability to socialize with colleagues	0.569		
Spaces for exercise, relaxation, or meditation	0.569		
Appreciation and recognition from superiors		0.800	
Business relations		0.798	
Reward from superiors		0.773	
Support system from colleagues or mentors		0.658	
Equality in development and treatment		0.536	
Noise		0.515	
Salary		0.508	
Workplace aesthetics			0.832
Work creativity			0.805
Feeling of giving			0.747
Space comfort			0.701

Extraction method: Principal Component Analysis; Rotation method: Varimax with Kaiser Normalization. The items “Respect from superiors”, “Respect from colleagues”, and “Possibility of temperature adjustments in the space” have been removed because they loaded on more than one component.

**Table 3:** Descriptive statistics and reliability indicators of the study variables.

	N Items	Cronbach’s a	M	SD	Median	Skewness	Kurtosis
QOL Domain Scores (0–100)							
Physical health	7	0.736	72.17	15.75	75.00	−0.545	−0.213
Psychological health	5	0.741	64.58	15.31	66.67	−0.616	0.221
Social relationships	3	0.847	68.89	24.08	75.00	−0.813	0.014
Environment	8	0.756	58.72	15.54	59.38	−0.359	−0.176
Overall QOL	1	-	65.63	18.64	75.00	−0.501	0.713
Satisfaction from health	1	-	72.08	22.96	75.00	−0.493	−0.233
Working conditions and benefits	9	0.891	3.21	0.90	3.33	−0.324	−0.579
Work relationships, rewards, and compensation	7	0.840	3.42	0.76	3.57	−0.563	−0.104
Workspace and nature of work	4	0.869	3.64	0.82	3.75	−0.765	0.759
Job satisfaction	4	0.829	3.17	0.97	3.25	−0.122	−0.747
Personal beliefs and values	4	0.818	4.04	0.62	4.00	−0.500	0.889
Spirituality	6	0.621	3.01	0.70	3.08	−0.273	−0.059

**Table 4:** Pearson correlation coefficients between study variables.

	1	2	3	4	5	6	7	8	9	10
Physical health	--									
Psychological health	0.652 **	--								
Social relationships	0.544 **	0.464 **	--							
Environment	0.673 **	0.605 **	0.511 **	--						
Working conditions and benefits	0.066	0.059	0.217 **	−0.044	--					
Work relationships, rewards, and compensation	0.007	0.024	0.172	−0.018	0.473 **	--				
Workspace and nature of work	0.158	0.176	0.293 **	0.134	0.539 **	0.557 **	--			
Job satisfaction	0.377 **	0.17	0.319 **	0.504 **	0.077	−0.002	0.142	--		
Personal beliefs and values	0.332 **	0.595 **	0.304 **	0.282 **	0.234 **	0.145	0.377 **	0.113	--	
Spirituality	0.108	0.262 **	0.132	0.223 **	0.194 **	0.321 **	0.324 **	0.221 **	0.418 **	--

** Very strong correlation among variables (*p* < 0.01).

**Table 5:** MANOVA results of tests of between-subject effects of gender, age, marital status, department, and education level on QoL domains.

Source	Dependent Variable	Type III SS	Df	MSE	F	*p*	η^2^_p_
Corrected Model	Physical health	2863.914 ^a^	8	357.99	1.56	0.148	0.11
	Psychological health	4547.003 ^b^	8	568.38	2.74	0.009	0.18
	Social relationships	11,181.755 ^c^	8	1397.72	2.68	0.010	0.18
	Environment	4036.145 ^d^	8	504.52	2.52	0.016	0.17
Intercept	Physical health	361,283.10	1	361,283.10	1570.91	<0.001	0.94
	Psychological health	295,091.40	1	295,091.40	1420.64	<0.001	0.94
	Social relationships	293,787.22	1	293,787.22	563.79	<0.001	0.85
	Environment	243,657.41	1	243,657.41	1216.65	<0.001	0.93
Gender	Physical health	39.61	1	39.61	0.17	0.679	0.00
	Psychological health	118.42	1	118.42	0.57	0.452	0.01
	Social relationships	763.33	1	763.33	1.47	0.229	0.02
	Environment	447.38	1	447.38	2.23	0.138	0.02
Age	Physical health	1995.74	3	665.25	2.89	0.039	0.08
	Psychological health	1187.74	3	395.91	1.91	0.134	0.06
	Social relationships	2802.83	3	934.28	1.79	0.154	0.05
	Environment	1681.32	3	560.44	2.80	0.044	0.08
Marital status	Physical health	291.79	1	291.79	1.27	0.263	0.01
	Psychological health	659.86	1	659.86	3.18	0.078	0.03
	Social relationships	5032.72	1	5032.72	9.66	0.002	0.09
	Environment	190.44	1	190.44	0.95	0.332	0.01
Department	Physical health	139.89	1	139.89	0.61	0.437	0.01
	Psychological health	0.88	1	0.88	0.00	0.948	0.00
	Social relationships	2659.13	1	2659.13	5.10	0.026	0.05
	Environment	128.29	1	128.29	0.64	0.425	0.01
Education	Physical health	794.56	2	397.28	1.73	0.183	0.03
	Psychological health	1553.88	2	776.94	3.74	0.027	0.07
	Social relationships	1061.42	2	530.71	1.02	0.365	0.02
	Environment	888.58	2	444.29	2.22	0.114	0.04
Error	Physical health	22,308.40	97	229.984			
	Psychological health	20,148.52	97	207.717			
	Social relationships	50,545.84	97	521.091			
	Environment	19,426.14	97	200.269			
Total	Physical health	581,403.06	106				
	Psychological health	465,746.52	106				
	Social relationships	549,652.77	106				
Corrected total	Environment	394,560.54	106				
	Physical health	25,172.31	105				
	Psychological health	24,695.52	105				
	Social relationships	61,727.59	105				
	Environment	23,462.28	105				

^a^ R^2^ = 0.114 (Adj. R^2^ = 0.041), ^b^ R^2^ = 0.184 (Adj. R^2^ = 0.117), ^c^ R^2^ = 0.181 (Adj. R^2^ = 0.114), ^d^ R^2^ = 0.172 (Adj. R^2^ = 0.104).

## Data Availability

Not applicable.

## Abbreviations

healthcare professionals—HCPs; quality of life—QOL; Principal Component Analysis—PCA.

## Appendix A. Pilot Study

The following questions were asked in addition to the questions of the WHOQOL (Greek version):

(1) In your opinion, what do you understand by the term employee well-being?
(2) What sort of practices had implemented either the administration of the department or that of the university to promote employee wellbeing?
(3) If you must use words to define your individual well-being at work in the department, what would they be? Please explain.
(4) Do you have time for breaks at work in the department? Please explain.
(5) What are your views about training received within the department in the last 12 months? What would you like to suggest for improvement?
(6) How do you feel about having a coach or mentor within the workplace to support you? Please explain.
(7) Do you think relationships between administration and staff are harmonious? Please explain.
(8) What are your views about teamwork in the department?
(9) What are your views about recruitment and selection strategies used in the department? Please explain.
(10) How satisfied are you with your job? Please explain.
(11) What are your views on being able to use your own initiative?
(12) Do you feel that your job is secure? Please explain.
(13) What are your views about being adequately compensated for work and effort that you put into your job?
(14) What are your views about the ethical code and reward policy used in the department? Please explain.
(15) What are your views on being involved in decision making in the department?
(16) If you were offered more money in another department/university would you think about changing job? Please explain.
(17) In terms of work-life balance, what are your views on work-life balance practices that should be implemented or enforced within the department?
(18) How have changes within the department affected your well-being? Please explain.
(19) How supportive are your colleagues and administration with non-work issues? Please explain.
(20) Can you describe what things you would like to see improved in the department to promote your well-being at work?

## Appendix B. Main Study

### Appendix B.1. Introductory Message

Dear colleagues,

You are invited to participate in an interdepartmental research activity of the Department of Dentistry and the Department of Nursing of the School of Health Sciences of the National and Kapodistrian University of Athens.

In your capacity as employees (faculty members, technical and administrative staff members, scientific associates, and postgraduate students) of these two departments, you are welcome to fill in the following questionnaire.

This questionnaire examines how you judge your quality of life, your health, and other aspects of your life at the present time. Please answer all the questions. You should read each question, think about, and evaluate your feelings by noting the number on the scale that gives the most appropriate answer for you. If you are unsure of the answer to a question, please choose the one you think is the most correct. Often, the most correct one may be the first answer you thought of. Please consider your own criteria, your own expectations, what gives you joy as well as what may concern you. We would like you to remember the last two weeks of your life.

The questionnaire is anonymous and in no way leads to the collection of personal data. Participation is voluntary and no reward is provided for your participation. Each employee can complete the questionnaire ONLY once. The completion of the questionnaire implies acceptance of the rules of personal data protection. The results of this study will be used for scientific publications and actions to improve working conditions in the departments involved.

Time to complete the questionnaire: 15 min.

The study is carried out under the auspices and approval of the Board of Ethics of the Department of Dentistry (No. 547/21 November 2022) and of the Department of Nursing (No. 426/9 January 2023).

### Appendix B.2. The Final Version of the Questionnaire

**Descriptive Part**

1. What is your gender? a. Male, b. Female, c. Other
2. How old are you? a. Up to 30 years old, b. 31–40 years old, c. 41–50 years old, d. 51–60 years old, e. 60 years and over.
3. What is your marital status? a. Single, b. Married, c. In cohabitation, d. Separated, e. Divorced, F. Widower, g. Other
4. Which category do you belong to? a. Faculty Member, b. Member of EDIP, c. EIB staff, d. Administrative staff of IDAX, e. IDOX administrative staff, F. IDAX technical staff, g. IDOX technical staff, h. Scientific associates (paid), i. Unpaid scientific associates, j. Postgraduate students.
5. If you maintain professional activity outside academic hours, what form does it take? a. Sole proprietorship, b. Sole proprietorship with 1 employee, c. Sole proprietorship with more than 2 employees, d. Company (member or director), e. Provision of services with block without individual headquarters, f. Hospital occupation, g. Provision with block without individual headquarters.
6. What is the highest education you received? a. Primary education, b. Secondary education, c. University, d. postgraduate degree
7. Do you have children? a. 0 none b. 1–2 children c. 3–4 children, d. 5 and over children
8. How many years do you receive a salary in the department? a. 1–10 years b. 11–20 years c. 21–30 years d. 31 and over.
9. What is your annual family income? Under 15,000, 15,001–25,000, 25,001 €–50,000 €, 50,001 €–100,000 €, 100,001 € and above
10. How is your health? Pretty poor (1)—Poor (2)—Neither poor nor good (3)—Good (4)—Very good (5)
11. Do you consider yourself currently ill? Yes… No…
12. If there is something wrong with you, what do you think it is? ________________________

**Instructions:** This questionnaire examines how you judge your quality of life, your health, and other aspects of your life. Please answer all the questions. If you are unsure of the answer to a question, please choose the one you think is the most correct. Often, the most correct may be the first answer you thought of. Please consider your own criteria, your own expectations, what gives you joy and what may concern you. We would like you to remember the last two weeks of your life. For example, thinking back to the last two weeks, one question might be:

How well are you able to concentrate?	Not at all 1	A little 2	A moderate amount 3	Very much 4	Extremely 5

You should circle the number that best fits how well you are able to concentrate over the last two weeks. So, you would circle the number 4 if you were able to concentrate very much. You would circle number 1 if you were not able to concentrate at all in the last two weeks.

**PART A**

Please read each question, think about, and evaluate your feelings, and circle the number of the scale that gives the most appropriate answer to each question.

		Very poor	Poor	Neither poor nor good	Good	Very good
Q1 (G1)	How would you rate your quality of life?	1	2	3	4	5
		Very dissatisfied	Moderately dissatisfied	Neither satisfied nor dissatisfied	Moderately satisfied	Very satisfied
Q2 (G4)	How satisfied are you with your health?	1	2	3	4	5

**PART B**

The following questions look at the extent to which you have had certain experiences or situations during the last two weeks.

		**Not at All**	**A Little**	**A Moderate Amount**	**Very Much**	**An Extreme Amount**
Q3 (F1.4)	To what extent do you feel that physical pain prevents you from doing what you need to do?	1	2	3	4	5
Q4 (F 11.3)	How much do you need any medical treatment to function in your daily life?	1	2	3	4	5
Q5 (F4.1)	How much do you enjoy life?	1	2	3	4	5
Q6 (F24.2)	To what extent do you feel your life to be meaningful?	1	2	3	4	5
Q7 (F5.3)	How well are you able to concentrate?	1	2	3	4	5
Q8 (F16.1)	How safe do you feel in your daily life?	1	2	3	4	5
Q9 (F22.1)	How healthy is your physical environment?	1	2	3	4	5

**PART C**

The following questions look at the extent to which you have had certain experiences or been able to do certain things during the last two weeks.

		**Not at All**	**A little**	**Μoderately**	**Mostly**	**Completely**
Q10 (F2.1)	Do you have enough energy for everyday life?	1	2	3	4	5
Q11 (F7.1)	Are you able to accept your bodily appearance?	1	2	3	4	5
Q12 (F18.1)	Have you enough money to meet your needs?	1	2	3	4	5
Q13 (F20.1)	How available to you is the information that you need in your day-to-day life?	1	2	3	4	5
Q14 (F21.1)	To what extent do you have the opportunity for leisure activities?	1	2	3	4	5
Q15 (F9.1)	How well are you able to get around?	Very poor	Poor	Neither poor nor good	Good	Very good

**PART D**

The next questions look at how good you felt or how satisfied you have been in various aspects of your life over the past two weeks.

		**Very Dissatisfied**	**Dissatisfied**	**Neither Satisfied nor Dissatisfied**	**Satisfied**	**Very Satisfied**
Q16 (F3.3)	How satisfied are you with your sleep?	1	2	3	4	5
Q17 (F10.3)	How satisfied are you with your ability to perform your daily living activities?	1	2	3	4	5
Q18 (F12.4)	How satisfied are you with your capacity for work?	1	2	3	4	5
Q19 (F6.3)	How satisfied are you with yourself?	1	2	3	4	5
Q20 (F13.3)	How satisfied are you with your personal relationships?	1	2	3	4	5
Q21 (F15.3)	How satisfied are you with your sex life?	1	2	3	4	5
Q22 (F14.4)	How satisfied are you with the support you get from your friends?	1	2	3	4	5
Q23 (F17.3)	How satisfied are you with the conditions of your living place?	1	2	3	4	5
Q24 (F19.3)	How satisfied are you with your access to health services?	1	2	3	4	5
Q25 (F23.3)	How satisfied are you with your transport?	1	2	3	4	5

The next question looks at how often you’ve had certain feelings over the past two weeks.

		**Never**	**Seldom**	**Quite Often**	**Very Often**	**Always**
Q26 (F8.1)	How often do you have negative feelings such as blue mood, despair, anxiety, depression?	1	2	3	4	5

**PART E**

The following questions address the factors that may affect your well-being in your workplace and the extent to which they are considered important. They relate to general working conditions prevailing over your work environment for the last 6 months.

**Q27. To What Extent Any of the Following Factors Affect Your Well-Being in Your Workplace?**	**Not at All**	**A Little**	**Μoderately**	**Mostly**	**Completely**
Working relations	1	2	3	4	5
Support system from partners or mentors	1	2	3	4	5
Noise	1	2	3	4	5
Work hours	1	2	3	4	5
Appreciation by superiors	1	2	3	4	5
Reward from superiors	1	2	3	4	5
Salary	1	2	3	4	5
Equality in development and treatment	1	2	3	4	5
Respect	1	2	3	4	5
Work creativity	1	2	3	4	5
Workplace aesthetics	1	2	3	4	5
Space comfort	1	2	3	4	5
Temperature settings in the workplace	1	2	3	4	5
Babysitting facilities	1	2	3	4	5
Possibility of continuing education in the same place	1	2	3	4	5
Possibility of a break in a special area	1	2	3	4	5
Observance of working hours	1	2	3	4	5
Flexibility of working hours	1	2	3	4	5
Possibility of a half-hour break for food	1	2	3	4	5
Healthy meals	1	2	3	4	5
Meditation gymnastics areas	1	2	3	4	5
Teamwork and socialization outside the workplace	1	2	3	4	5

**PART F**

The following questions are about your personal beliefs and values and how much they affect your quality of life. Once again, these questions refer to the last two weeks.

		**Not at All**	**A Little**	**Moderately**	**Mostly**	**Completely**
Q28 F24.1	To what extent do your personal values guide the life decisions you make?	1	2	3	4	5
Q29 F24.2	To what extent do you feel your life to be meaningful?	1	2	3	4	5
Q30 F24.3	To what extent do personal beliefs give you the strength to face difficulties?	1	2	3	4	5
Q31 F24.4	To what extent do personal beliefs help you understand life’s difficulties?	1	2	3	4	5
Q32 F24.5	Do you take responsibility for your reality?	1	2	3	4	5

**PART G**

The following questions are about spiritual pursuits that may be affecting your quality of life. These questions refer to religion, spirituality, and any other beliefs you may have. Once again, these questions refer to the last two weeks.

		**Not at All**	**A Little**	**A Moderate Amount**	**Very Much**	**An Extreme Amount**
Q33 F25.1	To what extent are you afraid of death?	1	2	3	4	5
Q34 F25.2	To what extent do you think there is a reason you live?	1	2	3	4	5
Q35 F25.3	To what extent have you been concerned about what happens after death?	1	2	3	4	5
Q36 F25.4	To what extent do you meditate?	1	2	3	4	5
Q37 F25.5	When faced with failure, to what extent do you continue to search for the meaning of your life?	1	2	3	4	5
Q38 F25.6	To what extent do you think a coach/mentor/spiritual guide would help you in your self-awareness and development?	1	2	3	4	5

**PART H**

The last session has two open-ended questions where you can explain your opinions more.

		**Please Briefly Write Your Suggestions**
Q39 F25.7	What would you like to change in your workplace to be more satisfied?	
Q40 F25.8	What would you like to change in your life to be happier?	

- How long did it take to fill this form out?  ………………………….. minutes
- Did someone help you to fill out this form? YES…………NO…………………

Thank you for your participation!
